# Supplementary material for: Protein primary structure correlates with calcium oxalate stone matrix preference
Source: PLoS One. 2021 Sep 23;16(9):e0257515. doi: 10.1371/journal.pone.0257515 (PMC8459966; doi:10.1371/journal.pone.0257515)
Supplement: Attachment — Submitted filename: Rebuttal letter July 2021.docx [file pone.0257515.s004.docx]

July 21, 2021

Rebuttal Letter for PLOS ONE article review.

**Title: Protein primary structure correlates with calcium oxalate stone matrix preference**

**Authors: Yu Tian, Matthew Tirrell, Carley Davis, Jeffrey Wesson,**

Dear Editors:

Thank you for your consideration of our manuscript and your many helpful comments. We have substantially revised the manuscript and are ready to resubmit. The technical comments have all been addressed to meet the standards and formats of your journal, and we have suppled both the marked-up copy and a clean copy per your request. A few specific items required comment in this letter as covered below. Some minor points from the reviewer specific comments have been addressed in our revised manuscript, but most of their comments were related to pertinent, though unanswered, questions related to our model. Comments to most of these questions have been detailed in our prior publications on the subject (references 8, 16, and 21). We will review these comments in this letter, but we did not modify the text. We have nothing new to add on these questions. This study is quite narrowly focused on the charged amino acid contents of urine and stone matrix proteins, and it was not designed to address any of these questions.

No modification is needed of the Reference list.

**Point by point comments to academic editor comments:**

1. The document has been reformatted to follow the PLOS ONE style requirements. Please notify me if any detail was missed.
2. We have created an Ethics statement for inclusion in both the manuscript (Methods section) and the submission form, but I reiterate here and in the Ethics statement that the studies reported in this manuscript used only the previously published data for COM stone matrix proteins (identity and abundance) from reference 8 and the previously published data for stone former urine (SFU) proteins (identity and abundance) from reference 21. Consequently, no new samples were acquired or tested for this manuscript, so technically no Ethics statement should be required. I have simply restated the Ethics statements from these two published reports to satisfy your request.
3. I included specific statements in my Ethics reply to explicitly restate that all individual identifying information was removed before publication. Only race, gender, and age data were associated with each reported sample, and these parameters would be insufficient to individually identify patients in our community of about 1.5 million people. I also noted that written informed consent was obtained for all newly recruited patients (stones or urine) under IRB approval.
4. We have removed the Acknowledgements section from the manuscript completely, as it only contained information related to funding. We wish to modify the Funding Statement to the following:

“Direct support for this work was supplied by the Department of Veterans Affairs through Merit Review funding from the Clinical Sciences Research and Development Division (grant number CX001491; JAW PI) and through the use of resources and facilities at the Clement J Zablocki Department of Veterans Affairs Medical Center, Milwaukee, Wisconsin. Also, indirect support was provided by a National Institutes of Health/ National Institute for Diabetes, Digestive, and Kidney Diseases (grant number DK82550; JAW PI).”

Please update the Funding Statement in the online submission form with the statement quoted above.

5. As noted above under item 2, all primary data on protein identities and abundances were extracted from data tables published in references 8 and 21 as described in our Methods section. These data were supplemented with amino acid content data from the widely used and freely accessible “Uniprot” database as cited in the text. The resultant complete tables of protein information for matrix preferring proteins and urine preferring proteins are included in Supplementary information as **S1 Table. Stone matrix phase preferring protein list** and **S2 Table. Urine phase preferring protein list**. There are no ethical or legal restrictions on sharing this information.
6. My ORCID ID is 0000-0002-2181-0323 and I will confirm it in the Editorial Manager.
7. See item #2 above. An Ethics statement was created in the Methods section as requested.
8. Captions were added to the Supporting information files as requested. References to this information were formatted as requested.

**Specific comments to reviewer’s questions:**

**Reviewer 1:**

“*The question is why protein protein interaction is favored*….” I have published several studies over many years focused specifically on characterizing the influence of proteins on calcium oxalate stone formation, and these experiments have supported the hypothesis that proteins likely assert primary influence over aggregation and growth of crystals (summarized in a review article, reference 14, in this manuscript). These studies have shown a 10,000 fold enhancement of inhibitory activities against nucleation, growth and aggregation of calcium oxalate crystal for selected relevant chemicals when comparing polymeric (30 or more residues bound in a chain structure) to monomeric forms of the same chemical, which is a direct justification for focus on the protein components. Proteins are also known to provide the dominant mass fraction to stone matrix, implying greater significance to their role in stone formation. This particular study was focused on identifying the underlying molecular interactions that likely lead to the collection of such a complex mixture of proteins in stone matrix; a mixture that is roughly duplicated by simply adding a strong polycation (polyarginine) to a solution of normal urinary proteins and studying the aggregates formed (reference 16 in this manuscript).

“*Is there a change in urinary profile of stone formers…?”* We have published a comparison the proteomic profiles in urine samples from healthy adults and stone formers (reference 21) finding relatively trivial differences between them, and certainly no evidence for the enrichment in stone former’s urine of either highly charged polyanions or highly charged polycations that we observed at increased abundance in stone matrix (reference 8). No studies have been published to date describing changes in the proteomic profile of urine proteins with time or environmental exposure that might help explain the stone matrix proteomic profile, though my current VA grant is funding research to look at urine proteomic profiles over time in both stone forming and healthy adults. Without an observed difference, we can only speculate on sources for a change in the urine proteome distribution.

We have provided the full names of proteins when they are first used in the text.

**Reviewer 2:**

1. The urine proteomic profiles of healthy adults and stone formers are almost completely overlapping/identical, but both urine profiles are different than what we observed in stone matrix (see references 8 and 21).
2. The majority of proteins observed in stone matrix in stones of all types are found in urine, but the relative amounts of individual proteins differ greatly between urine and matrix for calcium oxalate stones, as well as other stone types. Comparable data regarding the relative abundances of individual matrix proteins in other stone types have not been published yet. We do possess such data, and substantial differences can be seen between the stone matrix proteomic profiles observed in other stone types. At present, we cannot explain such differences, though we feel that they are important. We are still preparing this manuscript for publication.
3. Certainly, the proteins in human stones are of human origin, and cat stones contain cat proteins. Many urine proteins are secreted in the tubules, while others are filtered from the blood. Whether any of these proteins are derived from diet sources vs originating from normal physiologic processes within the body is currently unknown (unreported), and there are too many proteins involved in either urine or matrix to reasonably answer this question.
4. It is difficult to define a treatment before the mechanism is understood. Certainly, the presence of a large number of proteins in stone matrix that are normally found in intracellular or nuclear compartments argues for a cell injury process (likely tubular cells) as an originating event. We introduced this speculation in reference 8, but no further studies have been reported by us or others to address this possible mechanism, though hopefully my current VA funded investigation will provide some insight upon completion.
5. This question was addressed in the Discussion in reference 8. Current data cannot distinguish between a surge of highly charged polyanions and highly charged polycations creating protein aggregates which collect crystals vs a slow accumulation of these proteins from urine on existing crystals. The conversion of the very low abundance of the highly charged polyanions and polycations in urine to the observed distribution in stone matrix could be generated by the prolonged growth times of stones (months to years) through selective adsorption to crystal surfaces. A new experiment must be designed to test this question.
6. While I suspect that it is possible that pathological crystallization processes in other parts of the body may share protein characteristics with kidney stones, I am not aware of the appropriate protein data being available for crystal deposits in other organs to facilitate such a comparison.

I would like to publish the peer review process, since many questions have been posed and answered in the response to reviewers that may not fit in this manuscript specifically, but readers may find the discussions illuminating.

Sincerely,

Jeffrey A Wesson

jwesson@mcw.edu
